# Engagement is a necessary condition to test audit and feedback design features: results of a pragmatic, factorial, cluster-randomized trial with an embedded process evaluation

**DOI:** 10.1186/s13012-023-01271-6

**Published:** 2023-05-10

**Authors:** Nicola McCleary, Laura Desveaux, Justin Presseau, Catherine Reis, Holly O. Witteman, Monica Taljaard, Stefanie Linklater, Kednapa Thavorn, Gail Dobell, Cara L. Mulhall, Jonathan M. C. Lam, Jeremy M. Grimshaw, Noah M. Ivers

**Affiliations:** 1 Centre for Implementation Research, Clinical Epidemiology Program, Ottawa Hospital Research Institute, The Ottawa Hospital - General Campus, 501 Smyth Road, Room L1202, Box 711, Ottawa, ON K1H 8L6 Canada; 2 School of Epidemiology and Public Health, University of Ottawa, Ottawa, Canada; 3 Women’s College Research Institute, Women’s College Hospital, Toronto, Canada; 4 Institute of Health Policy, Management and Evaluation, University of Toronto, Toronto, Canada; 5 Institute for Better Health, Trillium Health Partners, Mississauga, Canada; 6 School of Psychology, University of Ottawa, Ottawa, Canada; 7 Institute for Health System Solutions and Virtual Care, Women’s College Hospital, Toronto, Canada; 8 Department of Family and Emergency Medicine, Laval University, Québec City, Canada; 9 Health System Performance, Ontario Health, Toronto, Canada; 10 Department of Medicine, University of Ottawa, Ottawa, Canada; 11 Department of Family and Community Medicine, Women’s College Hospital, Toronto, Canada; 12 Department of Family and Community Medicine, University of Toronto, Toronto, Canada; 13 ICES, Toronto, Canada

**Keywords:** Nursing home, Audit and feedback, Prescribing, High-risk medications, Cluster-randomized trial, Process evaluation

## Abstract

**Background:**

While audit & feedback (A&F) is an effective implementation intervention, the design elements which maximize effectiveness are unclear. Partnering with a healthcare quality advisory organization already delivering feedback, we conducted a pragmatic, 2 × 2 factorial, cluster-randomized trial to test the impact of variations in two factors: (A) the benchmark used for comparison and (B) information framing. An embedded process evaluation explored hypothesized mechanisms of effect.

**Methods:**

Eligible physicians worked in nursing homes in Ontario, Canada, and had voluntarily signed up to receive the report. Groups of nursing homes sharing physicians were randomized to (A) physicians’ individual prescribing rates compared to top-performing peers (the top quartile) or the provincial median and (B) risk-framed information (reporting the number of patients prescribed high-risk medication) or benefit-framed information (reporting the number of patients not prescribed). We hypothesized that the top quartile comparator and risk-framing would lead to greater practice improvements. The primary outcome was the mean number of central nervous system-active medications per resident per month. Primary analyses compared the four arms at 6 months post-intervention. Factorial analyses were secondary. The process evaluation comprised a follow-up questionnaire and semi-structured interviews.

**Results:**

Two hundred sixty-seven physicians (152 clusters) were randomized: 67 to arm 1 (median benchmark, benefit framing), 65 to arm 2 (top quartile benchmark, benefit framing), 75 to arm 3 (median benchmark, risk framing), and 60 to arm 4 (top quartile benchmark, risk framing). There were no significant differences in the primary outcome across arms or for each factor. However, engagement was low (27–31% of physicians across arms downloaded the report). The process evaluation indicated that both factors minimally impacted the proposed mechanisms. However, risk-framed feedback was perceived as more actionable and more compatible with current workflows, whilst a higher target might encourage behaviour change when physicians identified with the comparator.

**Conclusions:**

Risk framing and a top quartile comparator have the potential to achieve change. Further work to establish the strategies most likely to enhance A&F engagement, particularly with physicians who may be most likely to benefit from feedback, is required to support meaningfully addressing intricate research questions concerning the design of A&F.

**Trial registration:**

ClinicalTrials.gov, NCT02979964. Registered 29 November 2016.

**Supplementary Information:**

The online version contains supplementary material available at 10.1186/s13012-023-01271-6.

Contributions to the literature

- Head-to-head trials investigating ways to optimize audit and feedback impact are lacking but feasible within organizations already delivering feedback
- This head-to-head trial was unable to fully determine the effect of theory- and evidence-based variations in (i) the benchmark used for comparison (median vs. top quartile) and (ii) information framing (risk vs. benefit-framing), due to a lack of physician engagement
- The process evaluation indicated that emphasizing the risk of patient harms and using a benchmark more closely aligned with high-quality care is worth further exploration in contexts with high physician engagement
- This work demonstrates a successful partnership between researchers and health system stakeholders already delivering feedback at scale

## Background

Audit and Feedback (A&F) involves measuring a provider’s practice, comparing it to a benchmark, and relaying this information back to the provider to encourage change [1]. A&F leads to a median 4.3% absolute improvement in the provision of recommended care, with an interquartile range of 0.5 to 16% [1]. Therefore, whilst A&F is generally effective, there is considerable variation in effectiveness. Suggestions for optimizing A&F effectiveness have been published, highlighting specific areas where further research is needed [2]. A move away from two-arm trials evaluating A&F against usual care, and towards comparative effectiveness trials, would accelerate this process [3]. In addition, increasing the use of theory would help to establish the “active ingredients” of A&F and their mechanisms of effect [4]. We conducted a head-to-head trial investigating the impact on A&F effectiveness of theory-informed variations in two A&F design factors: the benchmark used for comparison and the framing of the A&F information.

In A&F reports, provider data are often compared to an average: for example, the median prescribing rate for physicians is a specific region. However, a higher benchmark might better represent high-quality care, and the level of the benchmark may impact motivation. Goal-Setting Theory predicts that setting specific goals, which are difficult but achievable, will have a greater impact on behaviour by increasing the effort made towards achieving the goal [5]. To our knowledge, few A&F trials have investigated the impact of different benchmarks. In line with Goal Setting Theory, greater improvements in practice are found when feedback recipients are compared to the median score achieved by the top 10% of peers (an “achievable benchmark”) versus comparison to the overall median [6–8].

The impact of information framing on providers’ and patients’ health-related decision-making and behavior has a long history of study [9–11]. Presenting the same information in different ways can lead to differing clinical decisions being made [9]. In the A&F context, the same underlying data can be used to indicate either the proportion of patients at risk of harms (e.g., prescribed high-risk medications) or the proportion of patients safe from the risk of harms (e.g., not prescribed high-risk medications). Social Cognitive Theory posits that outcome expectations (anticipated outcomes that may result from a behavior) predict behavior [12]. According to this theory, feedback that emphasizes risk to patients should encourage greater practice change by increasing physicians’ expectations that their patients are at risk of harm. To our knowledge, the impact of variations in A&F information framing on practice change has not been investigated.

A&F comparative effectiveness research may be best achieved by implementation scientists partnering with organizations already delivering A&F to create “implementation science laboratories” [13, 14]. This study involved such a partnership in the context of an existing A&F intervention. Such partnerships offer many advantages for the conduct of implementation research, including the opportunity to maximise sample size and fast-track recruitment processes by collaborating with teams who have already done significant work to engage practitioners in their intervention. Before study initiation, our health system partner had already achieved an A&F sign-up rate of 35% of eligible physicians.

The A&F intervention we evaluated aimed to support the appropriate prescribing of high-risk medications in nursing homes. Almost half of nursing home residents receive potentially inappropriate medications [15]. Some medications are considered potentially inappropriate in older adults due to age-related changes in drug metabolism and associated risks of adverse drug events [16, 17]. Nursing home residents prescribed potentially inappropriate medications have an increased risk of hospitalisation and death [18]. Population-based administrative data indicate that in our province (Ontario, Canada), older adults prescribed antipsychotics and nursing home residents prescribed benzodiazepines have a high risk of serious fall-related injuries [19, 20]. Such medications are often prescribed in nursing homes when not clinically indicated: for example, approximately 20% of nursing home residents in Canada are prescribed antipsychotics without a diagnosis of psychosis [21]. As such, implementation interventions aiming to reduce prescribing of high-risk medications in nursing homes where clinically appropriate are needed. A&F is effective in addressing over-use (i.e., for de-implementation problems) [1]. A previous version of the A&F intervention investigated here was associated with reduced antipsychotic prescribing [22]. We subsequently aimed to investigate the impact of two A&F design variables; specifically, we explored the impact of using different benchmarks for comparison and different approaches to information framing on the effectiveness of this A&F report in supporting appropriate reductions in high-risk medication prescribing in nursing homes.

## Methods

Our methods are described in detail in the published protocol [23] and are summarized here in accordance with the CONSORT Checklist for cluster randomized trials (Additional file 1). There were some deviations from the protocol, which are outlined below.

### Trial design

This was a 2 × 2 factorial, pragmatic, cluster-randomized trial with an embedded process evaluation. The trial is registered on ClinicalTrials.gov (NLM identifier: NCT02979964).

### Setting

This trial took place in the province of Ontario, Canada. Ontario Health (OH—formerly Health Quality Ontario at the time of the study), the provincial advisor on quality in healthcare, supports quality improvement through various initiatives. One such initiative is their “Practice Reports,” whereby confidential, aggregate feedback is offered to physicians across the province, combined with *change ideas* for quality improvement. These reports are populated using data from existing administrative health databases and are developed using the best evidence, established methods, and stakeholder advice. The senior author (NMI) established the Ontario Health Implementation Laboratory (OHIL), a partnership with OH to support the optimization of their A&F initiatives [13, 14]. This trial focused on OH’s “*MyPractice*: Long-Term Care” report (http://www.hqontario.ca/Quality-Improvement/Guides-Tools-and-Practice-Reports/Long-Term-Care) which provides physicians with feedback about their prescribing of medications for nursing home residents which potentially increase their risk of falls. At the time of the study, physicians had to opt-in to receive a feedback report and log into a system in order to access it.

### Participants

Eligible physicians were those working in the nursing home sector in Ontario who had (i) voluntarily signed up to receive their report prior to randomization and (ii) consistently had > 5 residents that they cared for in the nursing home setting (to allow for adequate data capture). The participating research ethics boards approved a waiver of consent with the provision of opt-out opportunities.

### Interventions and mechanisms of action

Full details of the history of the report and its re-design in preparation for this trial were reported previously [23]. The re-design process involved remote usability testing employing think-aloud methods to inform report optimisation [23]. Two report features were manipulated in this trial: (i) the benchmark used for comparison and (ii) information framing.

#### Manipulated feature 1—The benchmark

Previously, the report compared physicians’ data to provincial and regional averages. OH felt that a benchmark of the top 10% of peers used in previous research [6–8] may risk unintended discontinuation of appropriate medications. The top quartile was considered acceptable for the purposes of the trial while avoiding unnecessary harms to residents. Participants’ prescribing rates were therefore compared to either the median prescribing rate among Ontario physicians (Ontario median) or among physicians with the lowest prescribing rates (Ontario top quartile).

#### Manipulated feature 2—Information framing

We developed a “risk-framed” and a “benefit-framed” version of the report. The risk-framed version focused on the proportion of residents prescribed high-risk medication. Risk-framing was presented visually (a graph demonstrating the percentage of patients at risk, with red colouring), and in text form (“*n* additional/fewer resident(s) in my practice may be/are at increased risk associated with (medication)”). The benefit-framed version indicated the proportion of residents for whom high-risk medications were avoided, using a graph demonstrating the percentage of patients safe from risk, with green colour emphasis, and using the statement “*n* additional/fewer resident(s) in my practice may be/are safe from risks associated with (medication)”. Both versions were refined iteratively through user-testing [23].

**Fig. 1:**
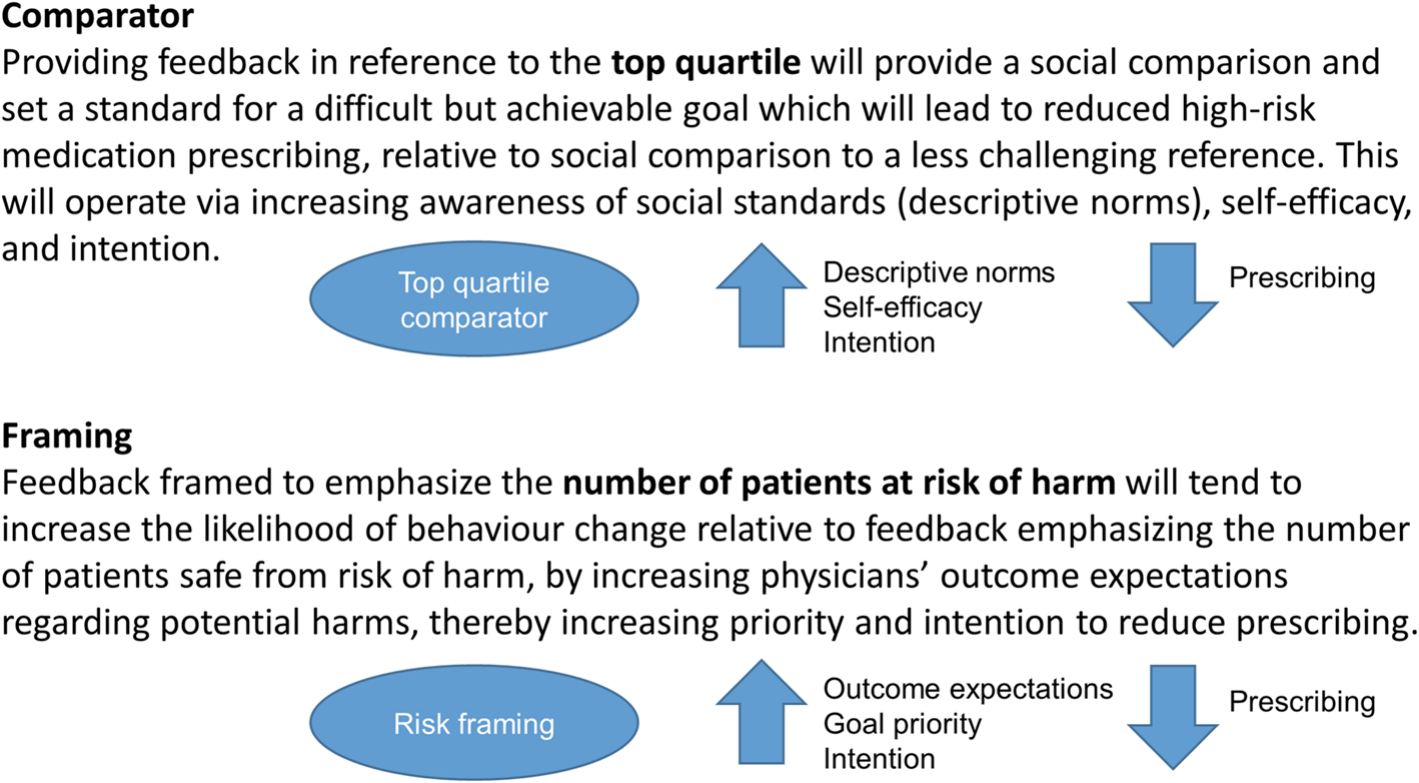
Proposed theory-informed mechanisms of action of the two factors varied in the Audit & Feedback report

Thus, four variants of the report were developed (excerpts included in Additional file 2): (i) Ontario median comparator with benefit-framing, (ii) Ontario top quartile comparator with benefit-framing, (iii) Ontario median comparator with risk-framing, and (iv) Ontario top quartile comparator with risk framing. Full details of our overall program theory are included in the protocol [23]. We hypothesized that greater improvements in practice would be achieved when feedback recipients were compared to the top quartile and when information was framed to emphasize risks of harm. Proposed theory-informed mechanisms of action are outlined in Fig. 1 [5, 24–27].

### Outcomes

The primary outcome was the mean number of central nervous system (CNS)-active medications per resident per month, with the primary endpoint for analysis being 6 months post-intervention. CNS-active medications included antipsychotics, opioids, benzodiazepines, and antidepressants (including tricyclic antidepressants and trazodone), consistent with the indicator used in the OH report. We selected this as the primary outcome to enable us to capture any prescribing changes directly influenced by the report indicators. We planned to assess antipsychotic and benzodiazepine prescriptions as secondary outcomes, as well as statin prescriptions (as a non-targeted control or “tracer outcome” [28]) [23]. However, we did not conduct these analyses due to poor report engagement (as described in the results).

### Data collection

In this pragmatic trial, we used provincial health administrative data to assess baseline characteristics and outcomes. Data were compiled from (1) the Ontario Drug Benefits database, which covers nearly all prescriptions in nursing homes; (2) the Canadian Institute for Health Information databases covering all inpatient hospitalizations and emergency department visits; (3) the Ontario Health Insurance Plan database, covering physician billings; (4) the Registered Persons Database covering demographic information; and (5) the Continuing Care Reporting System database for clinical and demographic information on nursing home residents collected using the Resident Assessment Instrument (RAI). A full RAI assessment completed by nursing home staff is legislatively mandated within 14 days of admission and updated annually or with a change in status; a quarterly RAI assessment is required every 92 days. RAI data were used to identify dates of admission and discharge to define the appropriate set of residents contributing to each time period. For each 3-month period under investigation, residents were assigned to a most responsible physician according to previously defined algorithms [29].

We used the RAI for demographic and clinical characteristics of residents, including clinical assessment scores (e.g., function scale, pain scale, depression rating score, aggressive behaviour score). We used Ontario Health Insurance Plan data to determine whether residents had a specialist consultation in the prior year by a geriatrician or psychiatrist. We used the Canadian Institute for Health Information datasets to assess whether residents had an emergency department visit in the prior year (using the National Ambulatory Care Reporting System database) and whether residents had a hospital admission in the prior year (using the Discharge Abstract Database). These databases provide complete population-level data for the variables of interest.

### Randomization

To prevent contamination due to physicians working across multiple homes, the unit of randomization was groups of one or more nursing homes sharing physicians. All eligible physicians were included in the clusters. An independent statistician randomized these clusters independently to the two factors (resulting in four experimental conditions), stratifying by a total number of nursing home beds in the cluster [30], using a randomly permuted block design of length four. The randomization list was provided to OH for the purposes of distributing the reports and was not accessible to any others on the research team besides the statistician.

### Sample size

We anticipated having approximately 160 clusters, with an average of 350 beds per cluster. In a 2 × 2 factorial design assuming no interaction and similar effects for each factor, a test of each intervention at 6 months in an ANCOVA design would achieve 90% power to detect an absolute mean difference of 0.3 in the primary outcome (i.e., a difference in the mean number of CNS-active medications per month of 3 versus 2.7). Based on previous data, we assumed a standard deviation of 4, an intracluster correlation coefficient of 0.05, a cluster autocorrelation of 0.8, and an individual autocorrelation of 0.9 [31].

### Blinding

Participants were not explicitly blinded, but the risks of this were felt to be minimal, given that the physician were not aware of the variations being tested nor the outcome measures. The analysts were blind to allocation status.

### Data analysis

Descriptive characteristics of nursing home residents included variables assessed as part of the RAI assessment: therefore, only those residents for whom a recent RAI assessment had been completed were included in the analysis of resident characteristics at baseline. Primary outcome analyses included the broader population of included physicians’ residents. All primary analyses were by intention-to-treat and compared the four arms. The primary outcome was analyzed using a general linear mixed effects regression model; time was specified as a continuous variable, and a common secular trend was imposed across all study arms with the effect of the intervention modelled as a slope deviation from the trend. The analysis adjusted for the size of each home (number of beds) as a fixed effect. A random intercept and slope for time were specified for the unit of randomization (group of homes). The primary comparison between the arms at 6 months post-intervention was estimated using least square mean differences, together with 95% confidence intervals. Factorial analyses were conducted as a secondary analysis because the traditional approach (i.e., an interaction test followed by dropping the interaction term if non-significant) can lead to bias in factorial trials [32]. OH provided data on report engagement (number of physicians who downloaded the report). Due to the poor engagement with the report (as described in the results), we did not conduct the additional analyses outlined in the protocol, including the economic evaluation.

### Process evaluation

Physicians who downloaded their report were sent an email invitation to complete a questionnaire which assessed the proposed mechanisms of action outlined in Fig. 1. The questionnaire included one item measuring each of these mechanisms in relation to prescribing three classes of high-risk medications and the tracer outcome (benzodiazepines, antidepressants, antipsychotics, and statins). Each item was scored using a five-point Likert scale. We compared construct scores for each of the trial factors using independent samples *t* tests. We planned to use mediation analyses to determine whether interventions worked through hypothesized pathways [23]: however, our small sample size precluded this investigation. All analyses were conducted using SPSS.

Questionnaire participants who indicated interest were invited to take part in a telephone interview. The interview topic guide focused on report use and ideas for improvement; prioritization of behavior change in relation to the prescribing indicators in the report; and the hypothesized mechanisms of action. Interviews were audio-recorded, then transcribed verbatim by an external third party. Analysis was conducted in NVIVO 10 and informed by the framework analysis method [33, 34]. An initial coding framework was developed, and constructs from the Consolidated Framework for Implementation Research [35] were added as this incorporated themes which developed from open coding. Refinement of themes involved study team discussions as necessary.

## Results

### Recruitment

Cluster and participant flow through the study is presented in Additional file 3. Before study initiation, 279 (35%) of 800 eligible physicians had voluntarily signed up to receive their report. On the 6th of December 2016, a report was released to 267 of these physicians at 152 clusters of nursing homes (12 physicians did not have enough data (< 5 patients)). The protocol reported 160 clusters randomized: however, for 8 potential clusters, OH was not able to provide a report to at least one physician within the cluster, so those clusters were not randomised. There were two further releases of the report during the study period: February 2, 2017, and April 26, 2017. These 267 physicians were randomized to one of the four versions of the report: 67 physicians in 38 clusters to arm 1 (median benchmark and benefit framing), 65 physicians in 37 clusters to arm 2 (top quartile benchmark and benefit framing), 75 physicians in 38 clusters to arm 3 (median benchmark and risk framing), and 60 physicians in 39 clusters to arm 4 (top quartile benchmark and risk framing). One physician from arm 2 was removed from all analyses as they activated their option to opt out of the evaluation. A total of 12,971 residents were included in our primary outcome analyses at the final follow-up (July 2017): 2904 (22%) in arm 1, 3334 (26%) in arm 2, 3989 (31%) in arm 3, and 2744 (21%) in arm 4.

### Report engagement

Of the 266 physicians analyzed, 76 (28.6%) in 60 clusters downloaded their report at the December 2016 release (19 (28.4%) physicians (13 clusters) in arm 1, 17 (26.6%) physicians (15 clusters) in arm 2, 23 (30.7%) physicians (16 clusters) in arm 3, and 17 (28.3%) physicians (16 clusters) in arm 4).

### Baseline resident characteristics

Baseline characteristics of nursing home residents for whom a recent RAI assessment had been completed in each arm and overall (December 1, 2016) are summarized in Table 1. Most residents were female (73%), aged between 78 and 95, and had been diagnosed with dementia (82%). Antipsychotics had been prescribed for 27% of residents, opioids for 26%, benzodiazepines for 14%, and antidepressants for 52%. Eighteen percent of residents had recently had a fall. Values for the baseline characteristics of included residents were similar across groups, except for the proportions of residents prescribed antidepressants (52% in arm 1, 57% in arm 2, 48% in arm 3, and 50% in arm 4).

**Table 1:** Baseline resident characteristics

	**Trial arm 1** **Median comparator & benefit framing (*n* = 2576)**	**Trial arm 2** **Top quartile comparator & benefit framing (*n* = 2958)**	**Trial arm 3** **Median comparator & risk framing (*n* = 3089)**	**Trial arm 4** **Top quartile comparator & risk framing (*n* = 2394)**	**Total (*n* = 11,017)**
Sex, *n* (%)					
Female	1847 (71.7%)	2169 (73.3%)	2285 (74.0%)	1696 (70.8%)	7,997 (72.6%)
Male	729 (28.3%)	789 (26.7%)	804 (26.0%)	698 (29.2%)	3,020 (27.4%)
Age					
Mean ± SD	86.36 ± 7.87	86.32 ± 7.92	86.40 ± 7.66	86.32 ± 7.78	86.35 ± 7.80
Charlson Comorbidity Index					
Mean ± SD	1.71 ± 1.56	1.73 ± 1.62	1.70 ± 1.58	1.76 ± 1.59	1.72 ± 1.59
Diagnosis, *n* (%)					
Psychosis	132 (5.1%)	166 (5.6%)	148 (4.8%)	112 (4.7%)	558 (5.1%)
Dementia	2127 (82.6%)	2326 (78.6%)	2594 (84.0%)	2011 (84.0%)	9,058 (82.2%)
Neither	317 (12.3%)	466 (15.8%)	347 (11.2%)	271 (11.3%)	1,401 (12.7%)
Level of function					
Mean ± SD	17.18 ± 7.05	16.26 ± 7.42	16.93 ± 7.20	16.93 ± 7.51	16.81 ± 7.30
Pain score					
Mean ± SD	0.37 ± 0.64	0.39 ± 0.65	0.39 ± 0.66	0.42 ± 0.69	0.39 ± 0.66
Depression score					
Mean ± SD	2.08 ± 2.36	2.43 ± 2.45	1.94 ± 2.32	1.90 ± 2.44	2.10 ± 2.40
Recent fall(s), *n* (%)	476 (18.5%)	518 (17.5%)	600 (19.4%)	420 (17.5%)	2,014 (18.3%)
Recent psychiatric consult, *n* (%)	230 (8.9%)	382 (12.9%)	393 (12.7%)	252 (10.5%)	1,257 (11.4%)
Recent geriatric consult, *n* (%)	125 (4.9%)	111 (3.8%)	174 (5.6%)	160 (6.7%)	570 (5.2%)
Recent restraint use, *n* (%)	90 (3.5%)	209 (7.1%)	142 (4.6%)	107 (4.5%)	548 (5.0%)
Aggressive behaviour					
Mean ± SD	1.44 ± 2.17	1.56 ± 2.22	1.50 ± 2.19	1.45 ± 2.26	1.49 ± 2.21
Prescriptions, *n* (%)					
Antipsychotics	668 (25.9%)	839 (28.4%)	797 (25.8%)	619 (25.9%)	2,923 (26.5%)
Opioids	655 (25.4%)	829 (28.0%)	758 (24.5%)	622 (26.0%)	2,864 (26.0%)
Benzodiazepines	355 (13.8%)	457 (15.4%)	389 (12.6%)	326 (13.6%)	1,527 (13.9%)
Antidepressants	1335 (51.8%)	1678 (56.7%)	1496 (48.4%)	1191 (49.7%)	5,700 (51.7%)

Analyses of baseline characteristics restricted to nursing home residents for whom a recent RAI assessment had been completed

### Effects of A&F variants on monthly number of CNS-active medications prescribed

Primary outcome analyses included the broader population of included physicians’ residents (i.e., not only those with a recent RAI assessment). The mean number of CNS-active medications prescribed per resident at the baseline month (December 1, 2016) and at the last follow-up time point (July 1, 2017) are displayed in Table 2.

**Table 2:** CNS-active medications per resident at baseline and follow-up

CNS-active medications per resident		Trial arm 1 Median comparator & benefit framing	Trial arm 2 Top quartile comparator & benefit framing	Trial arm 3 Median comparator & risk framing	Trial arm 4 Top quartile comparator & risk framing
Baseline	*n* residents	2817	3277	3768	2725
	Mean ± SD	1.12 ± 1.04	1.26 ± 1.10	1.07 ± 1.04	1.10 ± 1.07
At 6 months	*n* residents	2904	3334	3989	2744
	Mean ± SD	1.13 ± 1.02	1.28 ± 1.10	1.06 ± 1.02	1.10 ± 1.07

The general linear mixed effects regression model indicated there were no significant deviations from the secular trend in the monthly mean number of CNS-active medications in any of the arms (Table 3). In addition, there were no significant differences in the mean number of CNS-active medications prescribed per resident at 6 months post-intervention (July 1, 2017) in our analyses comparing the arms. The results of these analyses are presented in Table 4. In addition to the analyses by arm, factorial analyses were conducted: there were no appreciable differences in our findings (Additional file 4).

**Table 3:** Results of general linear mixed effects regression model predicting deviations from the secular trend in the monthly mean number of CNS-active medications

	**Estimate**	**95% CI**		**SE**	***p***
Intercept	1.219	1.126	1.312	0.047	< .001
Size of home (number of beds)	− 0.001	− 0.001	0.001	0.001	0.094
Pre-intervention secular trend slope	− 0.001	− 0.004	0.002	0.002	0.668
Trial arm 1 slope deviation from a secular trend	0.001	− 0.005	0.008	0.003	0.685
Trial arm 2 slope deviation from a secular trend	0.001	− 0.006	0.007	0.003	0.926
Trial arm 3 slope deviation from a secular trend	0.001	− 0.005	0.007	0.003	0.692
Trial arm 4 slope deviation from a secular trend	− 0.002	− 0.010	0.004	0.003	0.380

**Table 4:** Mean number of CNS-active medications prescribed across trial arms at 6 months post-intervention: pairwise comparisons

Comparison	Least square mean difference	95% CI for least square mean difference		*p*
Arm 2 vs. arm 1	− 0.007	− 0.056	0.041	0.761
Arm 3 vs. arm 1	− 0.001	− 0.047	0.045	0.964
Arm 4 vs. arm 1	− 0.030	− 0.080	0.020	0.232
Arm 3 vs. arm 2	0.006	− 0.038	0.051	0.779
Arm 4 vs. arm 2	− 0.023	− 0.072	0.026	0.355
Arm 4 vs. arm 3	− 0.029	− 0.076	0.017	0.217

### Process evaluation findings

Thirty-three physicians completed the questionnaire (6 in arm 1, 10 in arm 2, 9 in arm 3, 8 in arm 4). Descriptive statistics for the proposed mechanisms of action are reported by a trial factor in Table 5. The mean score for descriptive norms was significantly higher for those who received the median comparator than those who received the top quartile comparator, for antipsychotic (*t*(31) = 3.248, *p* = 0.003) and benzodiazepine (*t*(22.653) = 2.749, *p* = 0.012) prescribing. Those who received the top quartile comparator tended to score around the mid-point of the scale (neither agreeing nor disagreeing that their colleagues in other homes are appropriately adjusting their prescribing), while those who received the median comparator tended to score higher (agreeing, but not strongly agreeing, that their colleagues are appropriately adjusting their prescribing). No other significant differences were found between groups in any of the theoretical constructs for either factor.

**Table 5:** Descriptive statistics for proposed theory-based mechanisms of action

			**Intention** **Mean (SD)**	**Self-efficacy** **Mean (SD)**	**Outcome expectations** **Mean (SD)**	**Descriptive norms** **Mean (SD)**	**Goal prioritization** **Mean (SD)**
**Behavior: appropriately adjusting antipsychotic prescribing**							
**Factor**		***n***					
**Framing**	**Risk**	17	4.35 (0.49)	4.18 (0.81)	4.41 (0.51)	3.41 (0.80)	4.29 (0.59)
	**Benefit**	16	4.25 (0.68)	4.25 (0.86)	4.50 (0.63)	3.25 (0.68)	4.38 (0.72)
**Benchmark**	**Top quartile**	18	4.28 (0.58)	4.39 (0.78)	4.50 (0.51)	3.00 (0.69)**	4.39 (0.61)
	**Median**	15	4.33 (0.62)	4.00 (0.85)	4.40 (0.63)	3.73 (0.59)	4.27 (0.70)
**Behavior: appropriately adjusting benzodiazepine prescribing**							
**Factor**		***n***					
**Framing**	**Risk**	17	4.06 (1.03)	4.35 (0.70)	4.53 (0.51)	3.35 (0.49)	4.18 (0.73)
	**Benefit**	16	3.81 (0.66)	4.31 (0.79)	4.25 (0.78)	3.25 (0.45)	3.88 (0.62)
**Benchmark**	**Top quartile**	18	3.94 (0.73)	4.33 (0.69)	4.28 (0.75)	3.11 (0.32)*	3.89 (0.68)
	**Median**	15	3.93 (1.03)	4.33 (0.82)	4.53 (0.52)	3.53 (0.52)	4.20 (0.68)
**Behavior: appropriately adjusting antidepressant prescribing**							
**Factor**		***n***					
**Framing**	**Risk**	17	4.06 (0.83)	4.12 (0.70)	4.18 (0.81)	3.29 (0.59)	3.82 (1.07)
	**Benefit**	16	3.81 (0.54)	4.06 (0.57)	4.00 (0.63)	3.38 (0.50)	3.75 (0.78)
**Benchmark**	**Top quartile**	18	3.78 (0.65)	4.11 (0.68)	4.06 (0.64)	3.22 (0.43)	3.67 (0.97)
	**Median**	15	4.13 (0.74)	4.07 (0.59)	4.13 (0.83)	3.47 (0.64)	3.93 (0.88)
**Behavior: appropriately adjusting statin prescribing**							
**Factor**		***n***					
**Framing**	**Risk**	17	4.29 (0.59)	4.06 (0.83)	4.24 (0.66)	3.06 (0.56)	4.12 (0.78)
	**Benefit**	16	3.94 (1.0)	4.19 (1.05)	4.06 (0.77)	2.94 (0.57)	3.63 (1.09)
**Benchmark**	**Top quartile**	18	3.94 (0.94)	4.11 (1.13)	4.22 (0.73)	2.89 (0.47)	3.78 (1.06)
	**Median**	15	4.33 (0.62)	4.13 (0.64)	4.07 (0.70)	3.13 (0.64)	4.00 (0.85)

Scale: 1–5 Likert scale, strongly disagree-strongly agree (3 = neither agree nor disagree)

^**^p = .003 between groups (top quartile comparator vs. median comparator)

^*^p = .012 between groups (top quartile comparator vs. median comparator)

Five physician interviews were conducted. Two participants were women and three were men. Three had received the benefit-framed report with the top quartile comparator, and two had received the risk-framed report with the median comparator. Key themes are summarized below.

#### Participants’ intentions to adjust their prescribing were high before receiving the report

All participants highlighted their pre-existing intention to review medications and appropriately adjust their prescribing. Some also acknowledged that the report may have enhanced this. This corresponded to the CFIR domain *Characteristics of individuals (other personal attributes)*.

“Yeah so whether I look at this report or not I know for myself that I need to minimize the use of antipsychotic drugs, that’s there all the time.” (LTC4, risk, median)

“I think the idea of the report is for me a really good one because I think it’s just, this is your performance and, you know, can you do better? Like it sort of makes you look at it and think yeah can I do better… is there 1 or 2 people that I can get off these medications?” (LTC5, benefit, top quartile)

#### A comparator representing a higher target has the potential to influence prescribing behaviour change, if physicians identify with it

Participants indicated that comparing their performance to others was a key motivation for using the report. Participants who received the median comparator and those that received the top quartile comparator (a higher target) indicated that they aimed to achieve similar prescribing rates to the comparator. It also appeared that efforts to adjust prescribing were reduced when the comparator was reached or was close: this indicates a potential coasting effect (if physicians are close to the comparator, they may not prioritise it).

“When I’m at the 75th percentile or better, you know, I maybe don’t put as much emphasis on it” (LTC1, benefit, top quartile)

“The useful information for me is that either I am using less or I’m using the same as others in the, in Ontario… that’s good enough” (LTC4, risk, median) 

However, problems with identification with the comparator may negatively impact this. Participants emphasised that their prescribing rates should be considered in the context of the behavioural profile of their residents and that generalized comparators are not always appropriate. Participants discussed at length how their prescribing rates are reflective of the interaction between individual patient characteristics and the facility in which they reside. Various alternative comparators were suggested: for example, comparators based on the proportion of residents with certain cognitive/behavioural scale scores, or similar units (such as the presence of a secure unit). This corresponded to the CFIR domain *Implementation process (reflecting and evaluating)*.

“My ratio of aggressive behaviours double everybody else’s… so my antipsychotic use is a little higher, which isn’t surprising… Then the comments are, how do you de-prescribe? Well you know what I have a different unit is what my answer is… you can’t rate a percentage of antipsychotic use unless you’re looking at the population I’m dealing with.” (LTC2, benefit, top quartile)

#### Benefit-framed feedback is not immediately actionable and impedes report usability

Those receiving the benefit-framed report were vocal about the framing and found it difficult and time-consuming to visualize and interpret their data. They preferred a risk-framed report as this format matches other reports they receive and is viewed as more practical. Benefit framing therefore appears to decrease report usability. This corresponded to the CFIR domain *Intervention characteristics (complexity; design quality and packaging).*

“So how many of my residents are safe from the risks of falls associated with benzos? …you have to think about it a little bit more… if my percentage is lower that’s not good… I almost prefer the other way… because that’s the way it’s reported in our PAC meetings and it’s reported in CIHI that way… I think the negative has more impact… it’s a little bit easier to visualize.” (LTC5, benefit, top quartile)

#### Indicator selection may have hindered behaviour change efforts

Participants stated that of the three indicators, responding to the antipsychotic medication indicator by appropriately adjusting their prescribing was more of a priority than responding to the benzodiazepine indicator. Prioritisation of the “three or more specified CNS-active medications” indicator (i.e., the trial primary outcome) was rarely discussed. Participants expressed challenges with interpreting this indicator, specifically with identifying which medications were included. This precluded their ability to make sense of the data in order to influence behaviour change. This corresponded to CFIR domains *Characteristics of individuals (other personal attributes)* and *Intervention characteristics (complexity)*.

“The 3 or more specified I have to admit I don’t know where it’s specified. I don’t know which drugs they’re talking about.” (LTC2, benefit, top quartile)

#### Physicians value the report and suggested enhancements to help them monitor and discuss progress on improvement efforts at specific facilities

Participants noted that in summarizing prescribing data over a period of time, the report provides data not otherwise available and complements individual resident data already available. Some noted that the report informs discussions with other nursing home team members by “armoring” them with information. However, all participants practiced in more than one nursing home, and it was felt that discussions would be better facilitated if the report included data for all the participants’ facilities separately. This would allow for tailoring of prescribing adjustment efforts to different facilities (for example, taking the presence of a locked unit into account), and therefore enhance their ability to monitor progress with these efforts, and to apply the lessons learned and progress made at one facility to another. This corresponded to CFIR domains *Intervention characteristics (relative advantage)* and *Implementation process (reflecting & evaluating)*.

“Well I think it gives me some ammunition… the reports let me know what’s going on, how I’m comparing with the community and, sometimes pharmacists come up with ideas that are based on statistics… I like to have my own statistics… it’s a way to stimulate discussion.” (LTC3, risk, median)

“We have a protocol at (PRACTICE 2) where we are actually trying to discontinue or decrease the use of antipsychotic drugs in dementia patients… And I would love to know what my practice is there… But I don’t have that information… I could show them at (PRACTICE 2) that is why I’m using less there, because of the process we have in place.” (LTC4, risk, median)

## Discussion

### Summary of findings

We investigated the impact of variations in the benchmark used for comparison, and in information framing, on the effectiveness of A&F. In accordance with theory and evidence, we hypothesized that greater improvements in practice would be achieved when feedback recipients were compared to the top quartile rather than the median of their peers and when information was risk-framed rather than benefit-framed. There were no significant differences in the monthly mean number of CNS-active medications prescribed per resident over 6 months pre- and post-intervention in any of the four arms, or between arms at 6 months post-intervention. However, engagement with the report amongst those who signed up was poor, such that fewer physicians than anticipated were exposed to the design feature variations. In addition, the mean number of CNS-active medications was relatively low across all arms at baseline (ranging from 1.1 to 1.3), indicating that there was little room for improvement in the primary outcome. As a result, we did not proceed with some of the planned analyses, including the economic evaluation.

Importantly, the process evaluation revealed that both factors minimally impacted the proposed underlying mechanisms, which may also help to explain the lack of effects. However, benefit-framed feedback was not perceived as actionable, and physicians described aiming to align their practice with the top quartile, indicating that risk framing and a top quartile comparator still have the potential to achieve change.

### Interpretation and implications for the design and delivery of A&F

Around 35% of eligible physicians signed up for the report. However, under 30% of the physicians who signed up subsequently downloaded their reports. We therefore could not answer our relatively intricate research questions due to a lack of engagement. Previous work with this report showed that antipsychotic prescribing was reduced only for those who signed up for and downloaded their report [22], thereby demonstrating the requirement for adequate engagement before an A&F report can achieve impact. In addition, those who signed up were already “high achievers” (on average prescribing only 1.1 to 1.3 CNS-active medications per resident). Suggestions for optimizing A&F include recommendations to address barriers to engagement and to focus on behaviours with room for improvement [2].

Further research to establish strategies most likely to enhance engagement, particularly with physicians who may be most likely to benefit from feedback, would advance the science and practice of A&F. Clinical Performance Feedback Intervention Theory (CP-FIT) proposes that “pushing” A&F to providers rather than requiring the “pulling” of A&F, clearly demonstrating the potential benefits of the A&F, and targeting providers with positive attitudes to feedback all serve to enhance engagement and thereby A&F effectiveness [36]. At the time of this study, physicians had to log in and download their report (i.e., a “pull” process), which may have been a barrier to engagement. OH has since updated its procedures such that the reports are now emailed directly to the physicians. Future work could focus on designing and evaluating strategies aiming to clearly delineate feedback benefits and encourage positive feedback attitudes. These mechanisms could be targeted through a combination of behavior change techniques embedded within broader implementation strategies [37–39]. For example, information about various positive consequences that result from using A&F to inform practice improvements can be embedded within educational outreach visits or materials [39]. Positive feedback attitudes may be enhanced through discussions with credible sources (i.e., individuals or groups whom the target providers believe to be trustworthy) who themselves have positive attitudes to feedback. This can be incorporated into educational outreach visits, communications from local opinion leaders, or through train-the-trainer strategies [39].

It is also worth noting that whilst most participants did not download their report, they did demonstrate some initial engagement by voluntarily signing up for the report in the first place. Whilst the strategies suggested above may help to sustain their engagement, there may also be other factors making it difficult for them to integrate the use of A&F into their day-to-day practice. In this case, it may be worthwhile to investigate the impact of supportive goal setting, action planning, problem-solving, and habit formation strategies designed to encourage continued review and use of feedback. In sum, future research should focus on evaluating the effectiveness of a range of strategies aiming to enhance both initial and sustained engagement with A&F interventions. 

In accordance with Goal Setting Theory [5], physicians receiving the report including the top quartile comparator described being motivated to meet this higher target. A&F guidance recommends that comparisons to others should be challenging but achievable [2]. Guidance for A&F comparators published during our trial recommends tailored performance comparisons such that feedback recipients can compare themselves to high-performing others that are relevant to them [40]. Patel and colleagues [41] provide an example of the impact of tailored comparators: physicians whose prescribing rate was below the median received the median comparator, those between the median and the 90th percentile received the top 10% comparator, and those in the top 10% received a congratulatory statement instead of a comparator. This feedback combined with an active choice prompt led to 19% more physicians prescribing statins when compared to the active choice prompt alone. Our process evaluation indicated problems with identification based on the sources of the comparator data rather than the level of the target. Participants were unsure of whether their colleagues in other homes were appropriately adjusting their prescribing and expressed preferences for a comparator reflecting a similar context to their own. Future A&F research should explore the differential impact of generic high targets, tailored targets, and contextually tailored comparators. 

A key hypothesis of CP-FIT is that feedback is more effective when it fits alongside existing ways of working [36]. CP-FIT also proposes that feedback requiring less work is more effective [36]. In line with this, process evaluation participants who received benefit-framed information vocalised a preference for risk-framed information, perceiving it as more compatible with current workflows, less complex, and more actionable.

The process evaluation also indicated that both factors minimally impacted our proposed mechanisms of effect. Most participants across all groups intended to adjust their prescribing, were confident in doing so, prioritized adjusting their prescribing, and believed that doing so would avoid unnecessary risks to their residents’ health. This may indicate that these constructs were not the key barriers preventing change [42]. Our qualitative findings support this interpretation since participants noted their pre-existing intention to review medications and adjust prescribing. Our qualitative work also indicated that other factors not addressed by the report were serving as barriers to change. Specifically, the presentation of aggregate data which was not split according to the different homes a physician worked in impeded discussions of the data with colleagues and opportunities for continuous monitoring and adjustment of improvement efforts at specific sites. Previous qualitative work focused on antipsychotic prescribing in Ontario nursing homes found that interventions which involve multiple team members may reinforce behaviour change efforts [43]. In addition, CP-FIT hypothesises that A&F is more effective when implemented in a setting where team members work together towards a common goal. Taken together, this indicates that the effectiveness of A&F in the nursing home context may be enhanced by the inclusion of site-specific data and by additional content or co-interventions which enable team discussion and support behaviour change in wider team members. Our qualitative work also indicated that participants may have had difficulties interpreting the “three or more specified CNS-active medications” indicator (i.e., the trial primary outcome) and identifying which medications were included. The use of a composite indicator may therefore have introduced a barrier to change. These findings support the recommendation that feedback developers incorporate an assessment of potential barriers to feedback use into their development process to help maximise the impact of feedback on practice change [2].

Since this trial, OH has updated the design and delivery of its report. The report is now directly emailed to physicians. The information is risk-framed, focusing on the number of residents prescribed high-risk medications. The median prescribing rate is provided as the comparator, and in addition, physicians’ prescribing rates are described in reference to their peers. Specifically, prescribing rates higher than the 60th percentile are noted and highlighted in red; prescribing rates between the 25th and 60th percentiles are noted and highlighted in yellow and prescribing rates lower than the 75th percentile are noted and highlighted in green. Prescribing rates are reported overall and for each home a physician works in. The CNS-active medications indicator is still included but no longer features on the summary page as a “headline” indicator. The impact of the updated report on prescribing rates will be the subject of future work.

### Strengths and limitations

This trial demonstrates a successful partnership between researchers and health systems stakeholders to pragmatically investigate the impact of variations in the design of A&F focused on a topic of clinical importance in our context and already delivered at scale. This is the first in a series of studies aimed at optimizing A&F being conducted within the Ontario Health Implementation Laboratory (OHIL) which is also part of the international Audit and Feedback MetaLab initiative aiming to develop a more cumulative science to better inform A&F practice [13, 14]. As one of the few theory-informed head-to-head trials testing variations in A&F, our work builds on published recommendations for advancing A&F science [3]. The inclusion of a concurrent process evaluation allowed us to explore reasons for the lack of effectiveness, which will inform future OHIL studies. Key limitations are the low download rate of the report and the potential for selection bias, given that participants were prescribing few CNS-active medications per resident. In addition, the different types of information framing were challenging to operationalize due to the direction of behavior change targeted (i.e., reduce, but not eliminate, prescribing), and the fact that some participants’ prescribing rates were above the comparator, while others were below (therefore requiring different phrasing). So as not to risk encouraging inappropriate deprescribing, the report highlighted the number of patients who “may be” at risk/safe from risks. Where participants were performing above the comparator, the phrasing “fewer resident(s) in my practice are at increased risk/are safe from risks” may have been difficult to interpret. Overall, our findings informed an updated design of the A&F report led by OH. In addition, our findings can inform subsequent A&F research investigating strategies for report engagement, testing tailored comparators, optimizing information framing to ensure actionability and synergy with workflows, and examining the impact of feedback discussions in team-based care environments.

## Conclusions

This head-to-head trial of A&F delivered at scale found no impact of variations in either the benchmark used for comparison, or the framing of information, on physician prescribing of CNS-active medications to nursing home residents. However, we could not fully answer our research questions due to a lack of engagement with the report. The process evaluation indicated that a comparator representing a higher target can encourage behaviour change if physicians identify with it. In addition, feedback framed to emphasize the potential risk to patients is more actionable and more compatible with current workflows. Those designing and delivering A&F should consider the actionability of their indicators, how their report fits with current workflows, and the use of a comparator which may more likely represent a difficult but achievable goal. A&F researchers should explore the impact on A&F effectiveness of strategies for enhancing engagement, different types of tailored comparators, and co-interventions to support behavior change within healthcare teams.

## Supplementary Information

**Additional file 1.** Study Checklist.

**Additional file 2.** Excerpts from the A&F reports.

**Additional file 3.** CONSORT flow diagram.

**Additional file 4.** Results of factorial analyses.

## Data Availability

Trial data access is governed by the policies at ICES. Process evaluation data are available from the corresponding author on reasonable request.

## Abbreviations

A&F: Audit and Feedback
CNS: Central nervous system
OH: Ontario Health
OHIL: Ontario Health Implementation Laboratory
RAI: Resident Assessment Instrument
