# Supplementary material for: Engagement is a necessary condition to test audit and feedback design features: results of a pragmatic, factorial, cluster-randomized trial with an embedded process evaluation
Source: Implement Sci. 2023 May 10;18:13. doi: 10.1186/s13012-023-01271-6 (PMC10173488; doi:10.1186/s13012-023-01271-6)
Supplement: Supplementary file 2 — Additional file 2. Excerpts from the A&F reports. [file 13012_2023_1271_MOESM2_ESM.docx]

*Testing audit and feedback design features: factorial trial*

**Engagement is a necessary condition to test audit and feedback design features: results of a pragmatic, factorial, cluster-randomized trial with an embedded process evaluation**

**Additional File 2:** **Excerpts from the A&F reports**


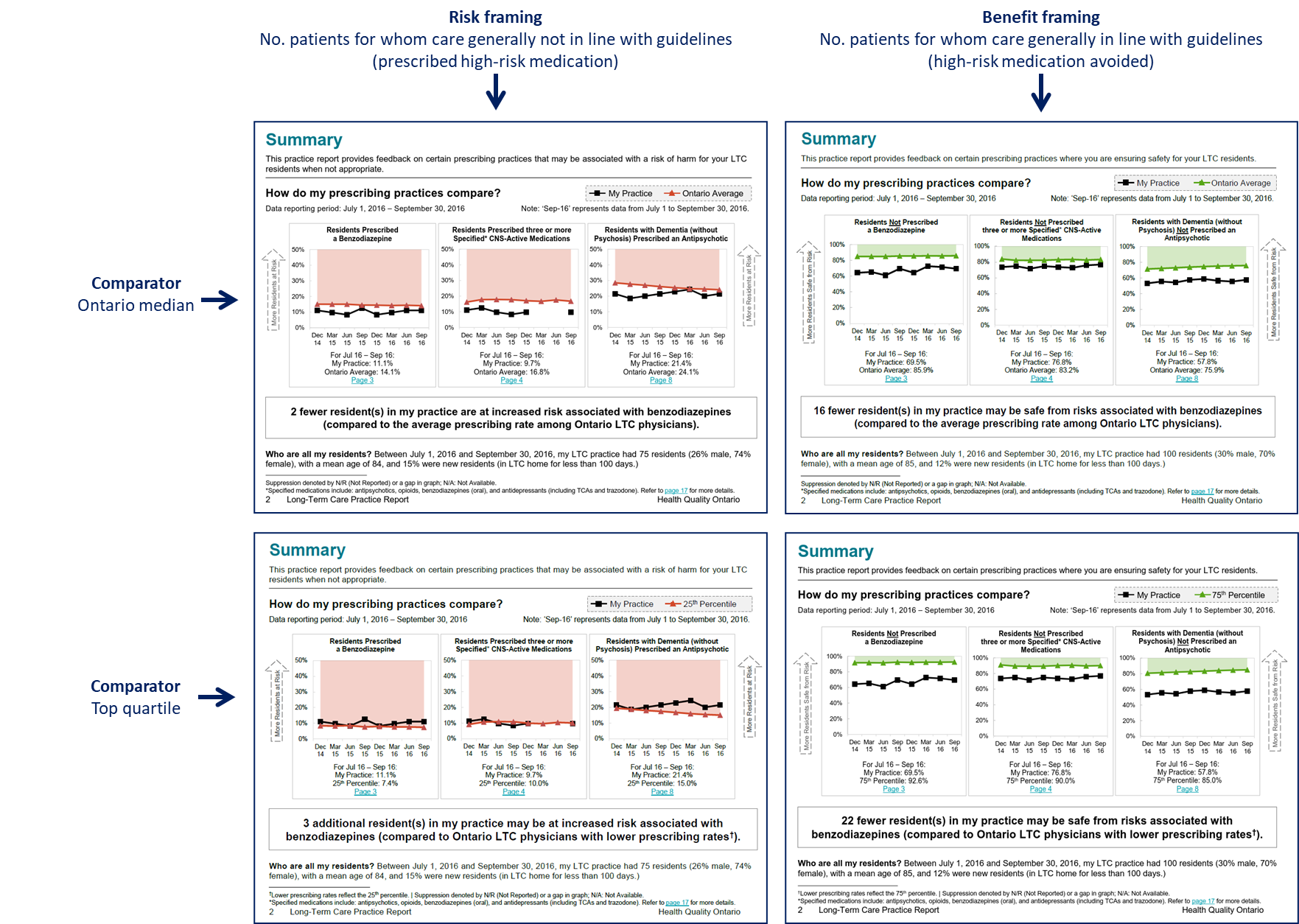


*Note: These excerpts are from example reports and therefore include mock data*
