# Supplementary material for: Engagement is a necessary condition to test audit and feedback design features: results of a pragmatic, factorial, cluster-randomized trial with an embedded process evaluation
Source: Implement Sci. 2023 May 10;18:13. doi: 10.1186/s13012-023-01271-6 (PMC10173488; doi:10.1186/s13012-023-01271-6)
Supplement: Supplementary file 3 — Additional file 3. CONSORT flow diagram. [file 13012_2023_1271_MOESM3_ESM.docx]

**Engagement is a necessary condition to test audit and feedback design features: results of a pragmatic, factorial, cluster-randomized trial with an embedded process evaluation**

**Additional File 3:** **CONSORT flow diagram**


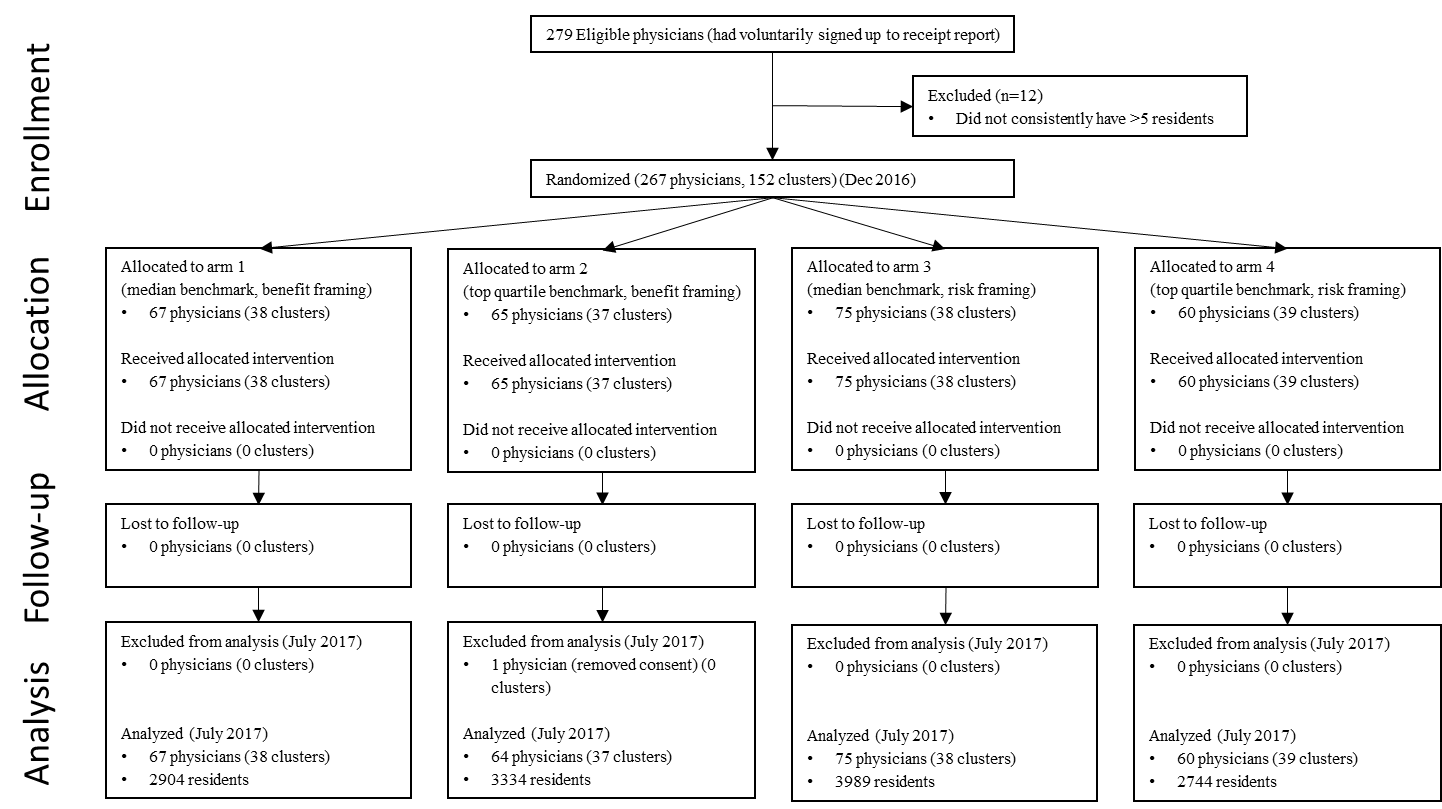


**e-Figure 2: CONSORT flow diagram**

Note: The protocol reported 160 clusters randomised: however, for 8 potential clusters, OHQ were not able to provide a report to at least one physician within the cluster, so those clusters were not randomised.
