# Supplementary material for: Engagement is a necessary condition to test audit and feedback design features: results of a pragmatic, factorial, cluster-randomized trial with an embedded process evaluation
Source: Implement Sci. 2023 May 10;18:13. doi: 10.1186/s13012-023-01271-6 (PMC10173488; doi:10.1186/s13012-023-01271-6)
Supplement: Supplementary file 4 — Additional file 4. Results of factorial analyses. [file 13012_2023_1271_MOESM4_ESM.docx]

**Engagement is a necessary condition to test audit and feedback design features: results of a pragmatic, factorial, cluster-randomized trial with an embedded process evaluation**

**Additional File 4:** **Results of factoeial analyses**

The mean number of CNS-active medications prescribed per resident at baseline (1st December 2016) and at the last follow-up time point (1st July 2017) are displayed in e-Table 1.

**e-Table 1. CNS-active medications per resident at baseline and follow-up for trial factors**

|  |  | **Factor 1: Benchmark** | | **Factor 2: Information framing** | |
| --- | --- | --- | --- | --- | --- |
|  |  | **Top quartile comparator** | **Median comparator** | **Risk framing** | **Benefit framing** |
| Baseline | N | 6,002 | 6,585 | 6,493 | 6,094 |
|  | Mean (SD) | 1.19 (1.09) | 1.09 (1.04) | 1.08 (1.05) | 1.20 (1.07) |
| Follow-up | N | 6,078 | 6,893 | 6,733 | 6,238 |
|  | Mean (SD) | 1.20 (1.09) | 1.09 (1.02) | 1.08 (1.04) | 1.21 (1.07) |

*Factor 1: Benchmark*

The general linear mixed effects regression model indicated that there were no significant deviations from the secular trend in the monthly mean number of CNS-active medications for either the top quartile or median comparator (e-Table 2).

**e-Table 2. Results of general linear mixed effects regression model predicting deviations from the secular trend in the monthly mean number of CNS-active medications for trial factor: benchmark**

|  | **Estimate** | **SE** | ***p*** |
| --- | --- | --- | --- |
| Intercept | 1.2269 | 0.04600 | <.0001 |
| Size of home (number of beds) | -0.00060 | 0.000289 | 0.0388 |
| Pre-intervention secular trend slope | -0.00052 | 0.001633 | 0.7525 |
| Top quartile comparator slope deviation from secular trend | -0.00123 | 0.002810 | 0.6613 |
| Median comparator slope deviation from secular trend | 0.000955 | 0.002740 | 0.7273 |

In addition, there were no significant differences in the mean number of CNS-active medications prescribed per resident at 6-months post-intervention (1^st^ July 2017) in our analyses comparing the comparator types (least square mean difference (95% CI) = 0.01531 (-0.01814, 0.04875), *p*=.3697).

*Factor 2: Information framing*

The general linear mixed effects regression model indicated that there were no significant deviations from the secular trend in the monthly mean number of CNS-active medications for either risk framing or benefit framing (e-Table 3).

**e-Table 3. Results of general linear mixed effects regression model predicting deviations from the secular trend in the monthly mean number of CNS-active medications for trial factor: information framing**

|  | **Estimate** | **SE** | ***p*** |
| --- | --- | --- | --- |
| Intercept | 1.2341 | 0.04687 | <.0001 |
| Size of home (number of beds) | -0.00055 | 0.000293 | 0.0606 |
| Pre-intervention secular trend slope | -0.00069 | 0.001632 | 0.6709 |
| Risk framing slope deviation from secular trend | -0.00062 | 0.002742 | 0.8222 |
| Benefit framing slope deviation from secular trend | 0.000735 | 0.002804 | 0.7932 |

In addition, there were no significant differences in the mean number of CNS-active medications prescribed per resident at 6-months post-intervention (1^st^ July 2017) in our analyses comparing the information framing types (least square mean difference (95% CI) = 0.009456 (-0.02384, 0.04275), *p*=.5778).
